# Supplementary material for: Effects of Follicle-Stimulating Hormone on Human Sperm Motility In Vitro
Source: Int J Mol Sci. 2023 Mar 31;24(7):6536. doi: 10.3390/ijms24076536 (PMC10095528; doi:10.3390/ijms24076536)
Supplement: Supplementary file 1 [file ijms-24-06536-s001.zip › ijms-2223251-supplementary.pdf]

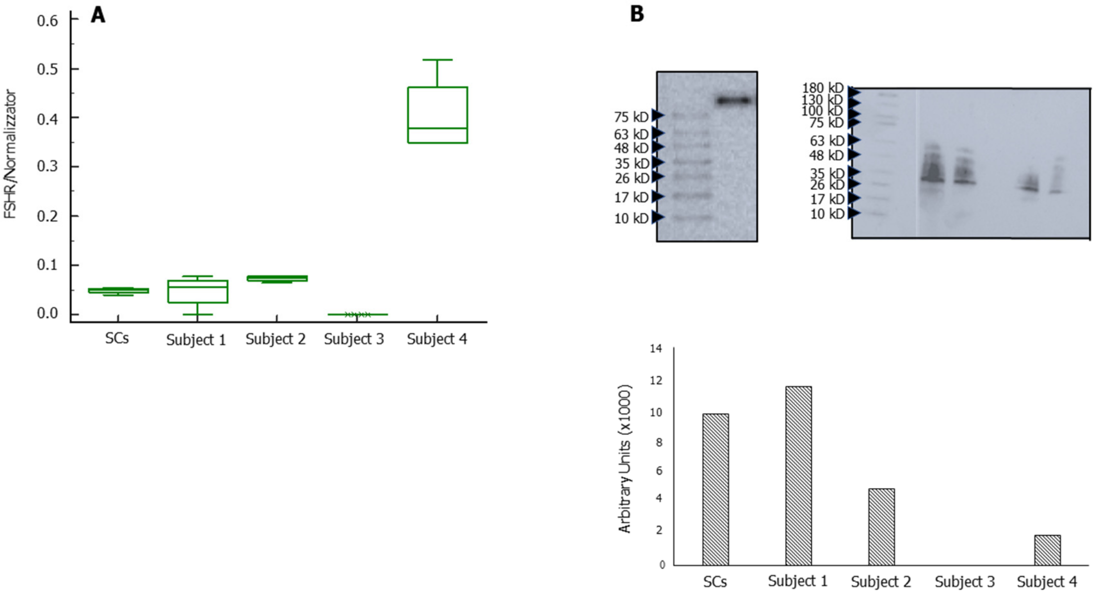

Supplementary Figure S1. Expression of follicle-stimulating hormone receptor (FSHR) mRNA and protein in human spermatozoa. The results of the RT-PCR analysis, shown on the left side (**A**), indicate the presence of FSHR mRNA in three out of four subjects. Immunoblots (right, upper panel) and densitometric analysis (right, bottom panel) (**B**) of FSHR in human spermatozoa confirm the expression of the protein in three out of four subjects. On RT-PCR, the FSHR mRNA was found in patients 1, 2, and 4. Western blot showed a 62–67 MW protein band in spermatozoa from the same patients. Sertoli cells (SCs) were used as a positive control. The subjects used for the experiment have been consecutively enrolled. Their sperm parameters are shown in detail in Supplementary Table S1. The data represent the mean ± standard deviation of three independent experiments, each performed in triplicate.

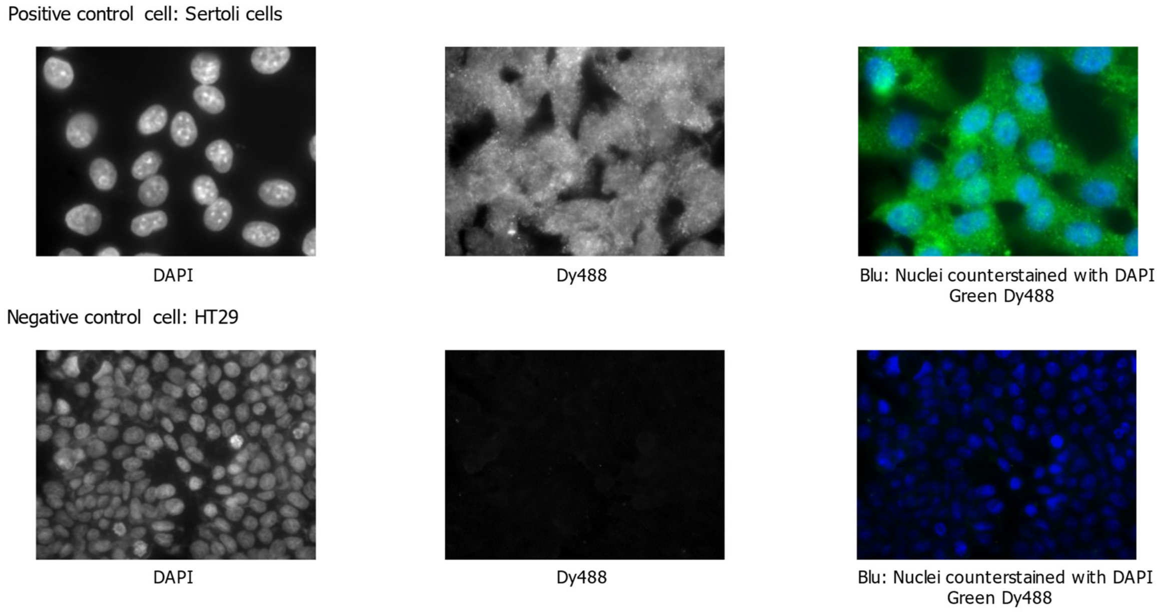

Supplementary Figure S2. Immunolocalization of the follicle-stimulating hormone receptor (FSHR) protein in porcine Sertoli cells and HT29 cells. The protein was detected by the FSHR antibody in Sertoli cells obtained from porcine testis, as positive control, while no signal was found in the negative control HT29 cells obtained from human colorectal adenocarcinoma.

Supplementary Table S1. Conventional sperm parameters of the subjects enrolled in this study for the assessment of sperm FSHR expression.

Subject ID	Sperm Concentration (Million/mL)	Total Sperm Count (Million/Ejaculate)	Progressive Motility (%)	Total Motility (%)	Normal Forms (%)	Leukocyte Concentration (Million/mL)
1	65	292.5	32	61	14	0.65
2	67	167.5	32	50	5	1.34
3	2	6	16	60	7	0.12
4	110	385	25	75	10	0
l.l.	>15	>39	>32	>40	>4	<1

FSHR, follicle-stimulating hormone receptor; l.l., lower limit according to the WHO criteria (2010).
